# Expressional Profiling of *TEX11*, *ESRα* and *BOLL* Genes in Yak under Different Feeding Conditions

**DOI:** 10.3390/biology10080731

**Published:** 2021-07-30

**Authors:** Qudratullah Kalwar, Min Chu, Anum Ali Ahmad, Lin Xiong, Yongfeng Zhang, Xuezhi Ding, Ping Yan

**Affiliations:** 1 Key Laboratory of Yak Breeding Engineering, Lanzhou Institute of Husbandry and Pharmaceutical Sciences, Chinese Academy of Agricultural Science, Lanzhou 730050, China; chumin@caas.cn (M.C.); anum2017@lzu.edu.cn (A.A.A.); xionglin@caas.cn (L.X.); zhangyongfeng_ying@163.com (Y.Z.); dingxuezhi@caas.cn (X.D.); 2 Department of Animal Reproduction, Shaheed Benazir Bhutto University of Veterinary and Animal Sciences, Sakrand 67210, Pakistan

**Keywords:** yak, concentrate supplementation, natural grazing, gene expression, *TEX11*, *ESRα*, *BOLL* genes

## Abstract

**Simple Summary:**

The yak (*Bos grunniens*) is regarded as one of the most magnificent domestic animals in the mountains of Asia, and it is well-adapted to the harsh environment of the Qinghai–Tibetan Plateau. Slow growth rate and low production and reproductive potential are the main limitations of yaks. It has been suggested that enhanced nutrition can improve reproductive efficiency in animals; however, this is still unclear for yaks. Hence, this study was designed to observe the effect of supplementary feeding on transcription and expression profiles of different genes related to reproduction. Such characterization under different feeding conditions can provide potential guidance for enhancement of the reproductive efficacy of yaks.

**Abstract:**

Previous studies have demonstrated that nutrition plays a crucial part in improving the reproductive potential of farm animals; however, there is currently no research on the transcription and expression profiling of genes in yaks under different feeding conditions. Therefore, this research was planned to compare the transcription and expression profiles of *TEX11*, *ESRα*, and *BOLL* in yaks under natural grazing with concentrate supplementation (NG + CS) and NG without concentrate supplementation. The transcription and expressional levels of *TEX11*, *ESRα*, and *BOLL* mRNA were explored from the testes of yaks using qPCR, Western blotting, immunofluorescence, and immunochemistry. The results of the qPCR illustrated that the transcription levels of *TEX11*, *ESRα*, and *BOLL* were upregulated in the NG + CS group compared to those in the NG group. Moreover, the results of the immunochemistry and immunofluorescence showed that the expression of TEX11, ESRα, and BOLL proteins increased after concentrate supplementation. Meanwhile, ESRα protein levels were lower in the testes and epididymides of yaks in the NG group than in those in the NG + CS group. Similarly, BOLL protein expression was higher in the testes and epididymides of the NG + CS group, but its expression was lower in the epididymides of the NG group. Furthermore, Western blotting showed that the molecular weights of ESRα and BOLL proteins were 64 kDa and 31 kDa, respectively. Finally, in the conclusion we summarize how a proper level of dietary energy supplementation can improve the reproductive potential of yaks by upregulating genes related to reproduction.

## 1. Introduction

Nutrition plays a key role in improving the reproductive potential of animals. Previous findings have determined that protein and energy supplementations are the main factors required to optimize the reproductive potential of animals [1]. Improper diet supplementation can cause a negative energy balance, resulting in the loss of body weight, increased postpartum interval to conception, delayed sexual maturity, and abnormal ovarian cyclicity through declining gonadotropin secretion, leading to infertility [2]. In addition, vitamins and minerals are crucial for a balanced diet; a lack of these nutrients may harm the reproductive efficacy of animals [3]. Reproduction is an energy-intensive process that has a significant impact on fat metabolism, the primary form of energy storage in animals [4]. During reproduction, animals mobilize their fat reserves; reduced or eliminated reproduction can increase lipid storage and lead to weight gain in many species [5,6]. Fatty acids and cholesterol are substrates for hormone synthesis, and increasing dietary fat may increase the levels of reproductive hormones (progesterone and prostaglandins). Fats may also have an effect on the reproductive system directly. As a result, fats may have effects that are independent of or additional to increased energy availability. According to an early study [7], feeding high-fat diets to cycling heifers and postpartum cows boosted progesterone production and the corpus luteum’s (CL) life span. In general, higher progesterone levels during the luteal phase lead to better fertility.

Testis-expressed gene 11 (*TEX11*) is a well-known germ cell-specific gene for spermatogonia-specific transcripts [8]. *TEX11* is expressed prominently in female and male germ cells and is crucial for meiotic progression and fertility [8]. Furthermore, its expression pattern reveals that *TEX11* exists in germ cells during spermatogenesis and testicular growth [9]. *TEX11* is exclusively expressed in the testis, and *TEX11* protein is observed in the cytoplasm and nuclei of type B spermatogonia. The highest level of *TEX11* is observed in zygotene spermatocytes, and a basal level is observed in late pachytene spermatocytes [10,11]. In addition, another study reported that male *TEX11* mice are infertile due to meiotic arrest [8].

Estrogen plays a significant role in reproduction [12]. The appearance of *ESRα* in the testis occurs very early during fetal development, and it is distributed in various testicular cells. The relationship between estrogen and the male reproductive system has been previously studied in several species [13,14]. *ESRα* has also been identified in the Sertoli cells of mature and immature rats [15]. It has also been reported that estrogen controls the function of Leydig cells during meiotic progression [16,17].

The RNA binding protein BOLL is an ancestral member of the deleted in azoospermia (DAZ) gene family, which plays an important role in maintenance of testicular function, testicular growth, and spermatogenesis, and loss of this gene may cause male dyszoospermia and infertility [18,19,20]. In the testes of infertile men, *BOLL* mRNA is significantly decreased and BOLL protein is completely absent compared to in healthy men [21]. Furthermore, BOLL protein is present in the cytoplasm of primary spermatocytes, and its absence is correlated with meiotic sperm arrest in a wide group of patients [22]. Yaks (*Bos grunniens*) are the only known bovine species that can live at high elevations (average altitude of 3000 m) [23]. Yaks can grow in and adapt well to the alpine grassland climate, and they can breed freely under difficult conditions on the plateau, such as low temperature, thin air, and in forage shortage [24]. Additionally, yaks are important livestock for the economy of the plateau region, providing local herdsmen with milk, meat, and transport [25]. However, yaks tend to exhibit slow growth, low production, and low reproduction efficiency and lose weight during long cold seasons (October to May) because of forage shortage, meaning that yaks only obtain limited energy. In addition, more energy is used to survive under low temperatures, causing yaks to suffer from energy stress for extended periods. When the energy from cured hay cannot meet the needs of yaks, some of the nutrient substances previously stored in yaks are decomposed to supply energy. As such, a traditional grazing system with low energy conversion efficiency causes yaks to be in a state of seriously inadequate energy and essential nutrient intake in winter [26]. Negative energy stress adversely affects reproductive performance in both sexes. In males, it reduces spermatogenic activity, while in females, it adversely impacts oogenesis, oocyte maturation, fertilization development, and implantation rate. Stress due to negative energy also affects the endocrine and biochemical conditions of male animals [27]. Similar changes have been observed in rams [28,29]. Heat stress also has endocrine effects, reducing the plasma luteinizing hormone (LH) level in bulls [30,31] and increasing the plasma testosterone level in boars [32]. The detection and evaluation of the deteriorating effects of negative energy stress on reproductive organs and cells could be helpful for designing measures to prevent these effects and improve reproductive functions. A previous study revealed that diet modifications may be beneficial for enhancing fertility [33].

We therefore hypothesized that concentrate supplementation would improve biological functions when animals are fed with concentrates compared to grass alone. Very little information exists regarding the expression profiling of genes under different feeding conditions. Hence, this study aimed to examine the effects of additional supplementation on transcription and expression profiles of different genes related to reproduction in a natural grazing with concentrate supplementation group and a natural grazing without concentrate supplementation group.

## 2. Materials and Methods

### 2.1. Animals

This study was conducted at the Key Laboratory of Yak Breeding Engineering of Gansu Province, Lanzhou Institute of Husbandry and Pharmaceutical Sciences. Sample collection was carried out in strict compliance with the Lanzhou Institute of Husbandry Animal and Pharmaceutical Sciences, China. Each animal was anesthetized before being slaughtered and every possible attempt was made to minimize suffering. The legal certificate number is SCXK (Gan) (2014-0002).

During this experiment, a total of 12 male yaks aged around 4 years old with the same body weights (250 ± 10 kg) were selected and randomly divided into two groups: the natural grazing with concentrate supplementation group (NG + CS) and the natural grazing without concentrate supplementation (NG) group, also known as the control (CO) group. Each group contained six randomly selected male yaks. The experiment was performed from October to February, and the animals were given a 14-day adaptation period for familiarization with the diets, facilities, and staff before the experiment. The dietary ingredients and nutrient composition were identified and are listed in Table 1. The experiment was performed in Haiyan County (100°250′ E, 36°550′ N) in Qinghai Province, China, from October to February. The NG + CS yaks were fed with totally mixed rations by stall feeding in breeding houses built in the same place. The animals were provided free access to water. Testis, epididymis, heart, fat, liver, kidney, and lung samples were collected from both groups. The extracted tissues were frozen for transport in liquid nitrogen and stored at −80 °C, and some samples were preserved in 4% formaldehyde for immunochemistry and immunofluorescence.

### 2.2. Extraction of RNA and Synthesis of Complementary DNA

Total RNA from each tissue was extracted using Trizol reagent (Tri Pure Isolation Reagent, Roche, Carlsbad, CA, USA), following the manufacturer’s instructions. The concentration and quality of RNA and DNA were examined using a Nano Drop™ Bio Photometer 2000 (Thermo Fisher Scientific, Inc., Waltham, MA, USA). The OD 260/280 nm values were within the appropriate range of 1.8–2.1, indicating that the purity of the RNA samples was good. Additionally, the 28S and 18S bands were clear and were not degraded by 1% agarose gel electrophoresis, indicating that the RNA integrity and quality were good and that follow-up tests could be carried out. Additionally, a Prime Script RT reagent kit with gDNA Eraser (Perfect Real Time) (TaKaRa Bio Inc., Shiga, Japan) was used for cDNA synthesis. After reverse transcription, cDNA was stored at −20 °C.

### 2.3. Primer Designing and PCR Amplification

National Center for Biotechnology Information (NCBI) Primer-BLAST (https://www.ncbi.nlm.nih.gov/tools/primer-blast/index.cgi?LINK_LOC=blastHome) [20] was used to design the primers (Table 2). A total amount of 25 μL PCR mixture, including 2 μL primers (forward and reverse), 12.5 μL GoTaq^®^ Green Master Mix, 2 μL cDNA, and 8.5 μL H_2_O (Promega, Madison, WI, USA), was used to check the amplification of the primers. PCR reactions were performed with initiation at 95 °C for 5 min, followed by 35 denaturation cycles at 95 °C for 1 min, annealing at 56–60 °C for 45 s and 72 °C for 1 min, final extension at 72 °C for 5 min, and storage at 4 °C for 5 min. Finally, 1% agarose gel was used to load the PCR products.

### 2.4. Exploration through Quantitative Real-Time PCR

The *Tex11*, *ESRα*, and *BOLL* mRNA isolated from tissues were quantified against the GAPDH housekeeping gene using qRT-PCR. The PCR reactions for qRT-PCR were carried out using SYBR Premix Ex TaqTM (TaKaRa Bio Inc., Shiga, Japan). PCR was performed with a Thermal Cycler Dice Real-Time System (Bio-Rad, Hercules, CA, USA) under the following cycling conditions: 95 °C for 30 s followed by 40 cycles of 95 °C for 5 s and annealing at 64.5 °C for 30 s.

### 2.5. Exploration through Western Blotting

Proteins from the samples were extracted as described previously [20]. Testis samples from both groups were homogenized in ice-cold RIPA buffer (25 mM Tris/HCl (pH 7.6), 1% sodium deoxycholate, 150 mM NaCl, 0.1% SDS, 1% Nonidet-P40, and 0.05 mM PMSF) and centrifuged at 15,000 rpm for 10 min at 4 °C. Subsequently, the concentration of total protein was evaluated using a BCA protein assay (Santa Cruz Biotechnology, sc-202389). Samples (40 μg protein per lane) were subjected to 10% SDS-PAGE gel electrophoresis and then transferred to PVDF membranes (Roche). Membranes were blocked with 5% milk powder in 1 × 9 phosphate-buffered saline (PBS) and 0.1% Tween 20 for 60 min, washed with PBS/Tween, and incubated overnight at 4 °C with primary antibodies, including anti-ESRα and BOLL (1:500 dilution; Abcam, Cambridge, UK) and anti-β-tubulin (1:1000 dilution; Santa Cruz Biotechnology). Membranes were then incubated for 2 h with an anti-rabbit secondary antibody, and bands were imaged using an ECL detection system (GE Healthcare Bio-Sciences, Pittsburgh, PA, USA). Finally, photographs were taken after exposure to the X-ray films.

### 2.6. Immunochemistry of *TEX11, ESRα and BOLL* Proteins

Immunohistochemical staining protocols for TEX11, ESRα, and BOLL proteins from previous studies were followed [34,35]. The testicles were deparaffinized and PBS was used to wash the slides to avoid any further endogenous peroxidase development. Tissue sections were treated at 37 °C for 15 min with 3% hydrogen peroxide in PBS. Slides were then blocked at ambient temperature for 1 h in 5% serum in PBS and incubated at 4 °C with anti-TEX11, anti-ESRα, and anti-BOLL antibodies (1:500 dilution; Abcam, Cambridge, UK) overnight. The slides were incubated at room temperature with HRP-conjugated anti-rabbit secondary antibody for 1 h after washing with PBS. Finally, images were taken using a microscope (Leica, Germany).

### 2.7. Immunofluorescence Analysis

Briefly, slides of deparaffinized tissues were incubated overnight at 4 °C with primary antibodies against TEX11, ESRα, and BOLL. Slides were subsequently washed in PBS, an analogous secondary antibody was added, and segments were incubated at 37 °C for 2 h. The segments were then washed again with PBS and diamino-2-phenylidole was used for nuclear staining. Finally, photos were taken using an Olympus microscope (BX53) and a camera (Olympus DP73, Olympus Corporation Company, Tokyo, Japan).

### 2.8. Statistical Analyses

The quantitative mRNA expression level of the target gene was calculated using the threshold cycle 2^−ΔΔCt^ method [36]. Hematoxylin and eosin (H&E) staining and histomorphometry images of testicular cross-sections were captured using Image View software (Sunny, Ningbo, China). The morphological parameters of randomly selected seminiferous tubules using H&E sections at 200× magnification were measured using Mv Image software (Sunny, Ningbo, China). The integral optical density of immunostaining for the proteins was calculated by analyzing four random 400× microscope magnification levels in independently replicated sections using Image-Pro Plus 6.0 software (Media Cybernetics, Rockville, MD, USA). In addition, statistically significant differences among the means were analyzed by analysis of variance (ANOVA) and *t*-tests (*p* < 0.05). Data are expressed as the mean ± standard deviation (SD).

## 3. Results

### 3.1. Expressional Profiling of *TEX11, ESRα*, and *BOLL* Genes through Quantitative Real-Time PCR

**Figure 1:**
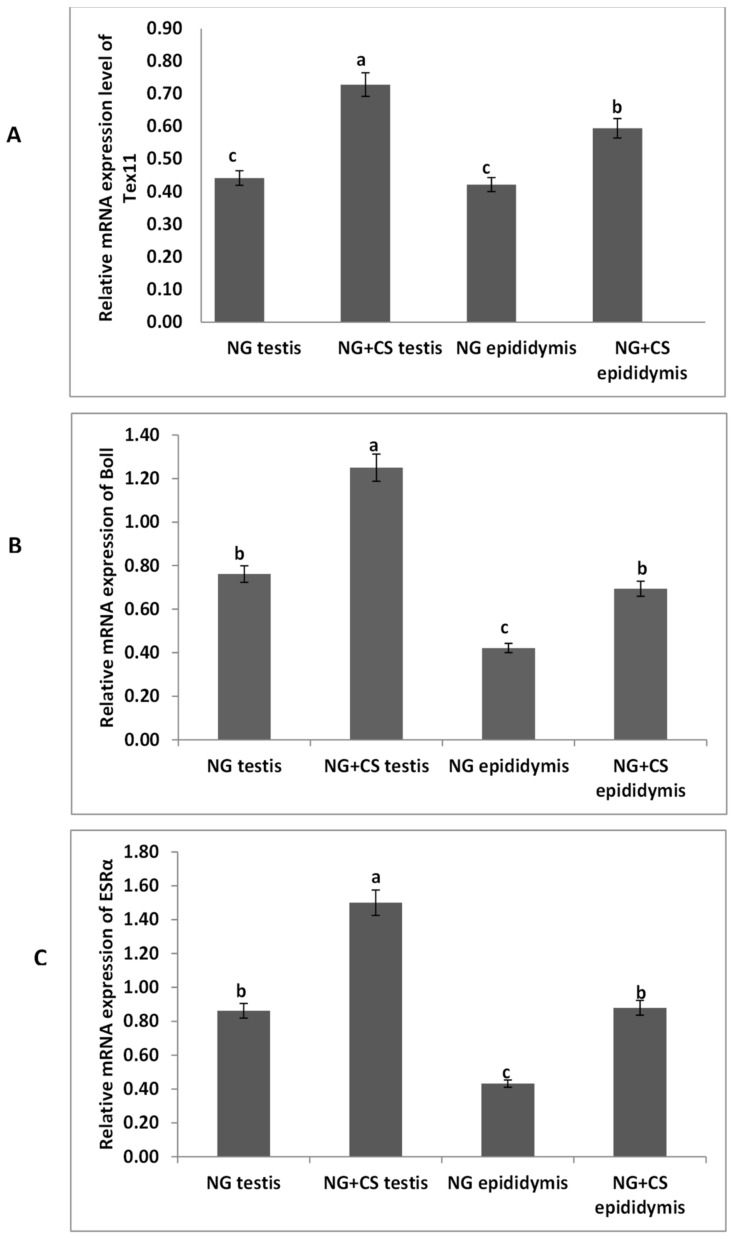
Expression analysis of *TEX11*, *BOLL*, and *ESRα* mRNA using qPCR: (**A**) *TEX11*, (**B**) *BOLL*, (**C**) *ESRα* mRNA. NG = natural grazing without concentrate supplementation group, NG + CS = natural grazing plus concentrate supplementation group. Different letters indicate a significant difference (*p* < 0.05).

The expression levels of the *TEX11*, *ESRα*, and *BOLL* from the yak tissues were evaluated by qPCR under different feeding conditions (Figure 1). Our qPCR results revealed that the expression of *BOLL* mRNA was highest in the testis, followed by the epididymis, for the treated group. Similarly, *TEX11* and *ESRα* expression levels were higher in the testis and epididymis of the treated group as compared to the control group.

### 3.2. Western Blotting Analysis of *ESRα* and *BOLL* Proteins

**Figure 2:**
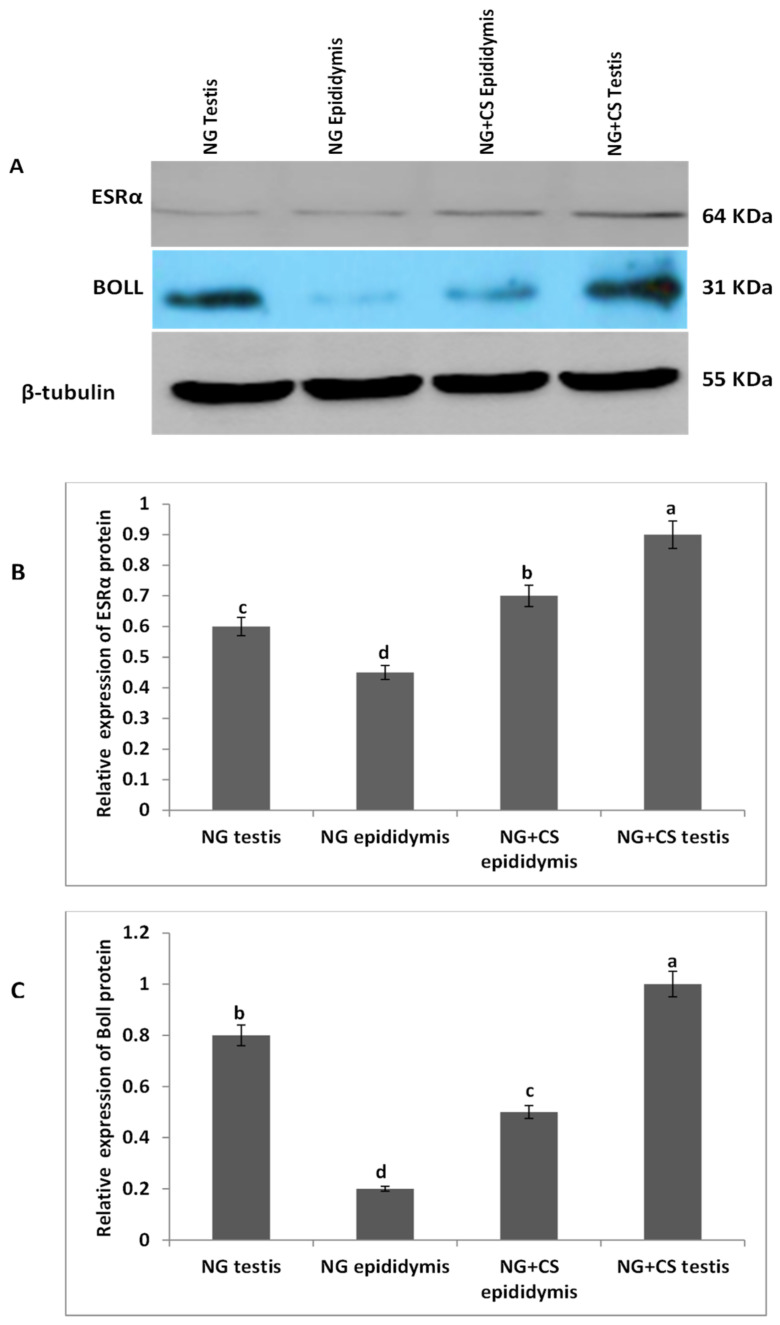
Characterization of ESRα and BOLL proteins in the testes and epididymis of Datong yaks with and without concentrate supplementation: (**A**) Western blotting of ESRα and BOLL proteins; and the full Western blotting of ESRα and BOLL proteins is shown in Figure S1; (**B**) relative expression of ESRα protein; (**C**) relative expression of BOLL protein; (1) testis without concentrate supplementation; (2) epididymis without concentrate supplementation; (3) epididymis with concentrate supplementation; (4) testis with concentrate supplementation; Beta tubulin was used as control. Protein levels were quantified using densitometric analysis. Significant variations are shown by different letters (*p* < 0.05).

Additionally, Western blotting was performed to evaluate ESRα and BOLL proteins in the testis and epididymis of Datong yaks with and without concentrate supplementation (Figure 2). Our Western blot results indicated that the expression of both proteins increased after concentrate supplementation. Moreover, ESRα protein was lower in the testes and epididymis of the group without concentrate supplementation than in the NG + CS group. Similarly, expression of BOLL protein was higher in the testes and epididymis of the NG + CS group, and lower expression was observed in the epididymis of the control group.

### 3.3. Immunohistochemistry of *TEX11, ESRα, and BOLL* Proteins

**Figure 3:**
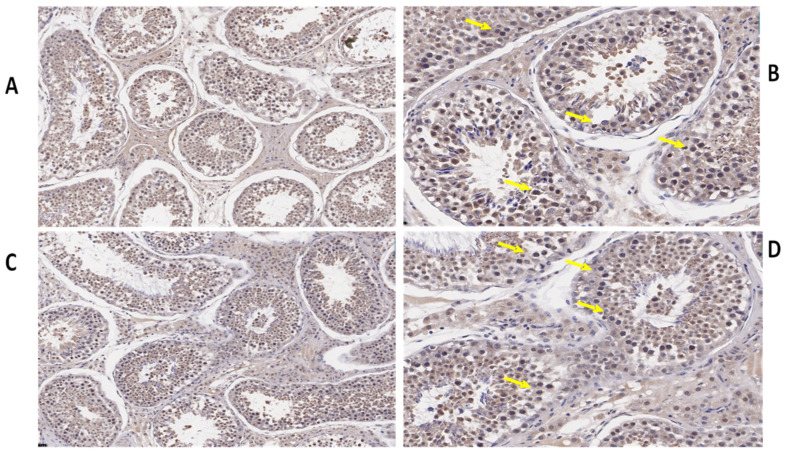
Immunohistochemical localization of TEX11 protein in the testes of yaks under different feeding conditions. Immunohistomestry indicated that TEX11 protein was present in both groups but positive cells (yellow arrow) were more prominent in the tubules of the concentrate supplementation group as compared to the group without concentrate supplementation: (**A**,**B**) natural grazing plus concentrate supplementation; (**C**,**D**) natural grazing without concentrate supplementation. Scale bar = 50 µm (**A**–**D**).

**Figure 4:**
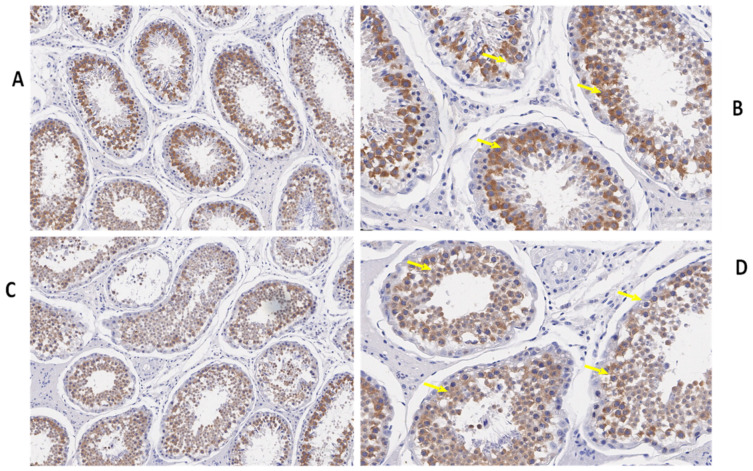
Immunohistochemical localization of BOLL protein in the testes of yaks under different feeding conditions. Immunohistomestry indicated that boll protein was present in both groups but positive cells (yellow arrow) were more prominent in the tubules of the concentrate supplementation group as compared to the group without concentrate supplementation: (**A**,**B**) natural grazing plus concentrate supplementation; (**C**,**D**) natural grazing without concentrate supplementation. Scale bar = 50 µm (**A**–**D**).

**Figure 5:**
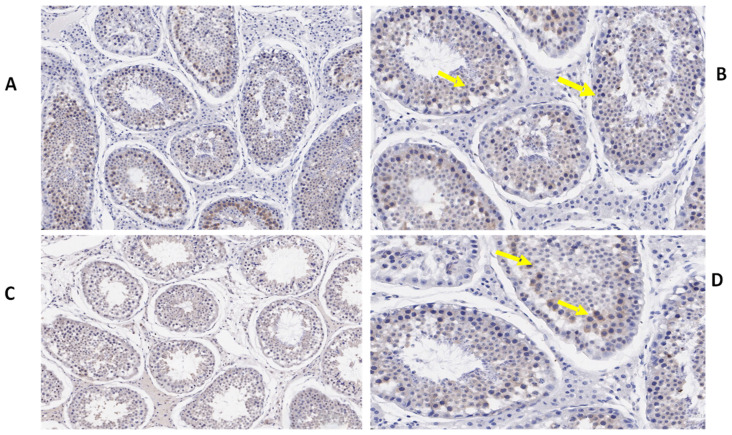
Immunohistochemical localization of ESRα protein in the testes of yaks under different feeding conditions. Immunohistomestry indicated that TEX11 protein was present in both groups but positive cells (yellow arrow) were more prominent in the tubules of the concentrate supplementation group as compared to the group without concentrate supplementation: (**A**,**B**) natural grazing plus concentrate supplementation; (**C**,**D**) natural grazing without concentrate supplementation. Scale bar = 50 µm (**A**–**D**).

We also determined the morphological differences in the groups with and without concentrate supplementation by using immunostaining analysis (Figure 3, Figure 4 and Figure 5). Immunohistochemistry revealed that TEX11 protein (Figure 3), BOLL protein (Figure 4), and ESRα protein (Figure 5) were present in both groups, but the numbers of positive cells (yellow arrow) were greater in the tubules of the NG + CS group than in the control group. Additionally, the integrated optical densities of TEX11, ESRα, and BOLL were significantly higher in the NG + CS group than in the control group (*p* < 0.05).

### 3.4. Immunofluorescence of *TEX11, ESRα and BOLL* Proteins

**Figure 6:**
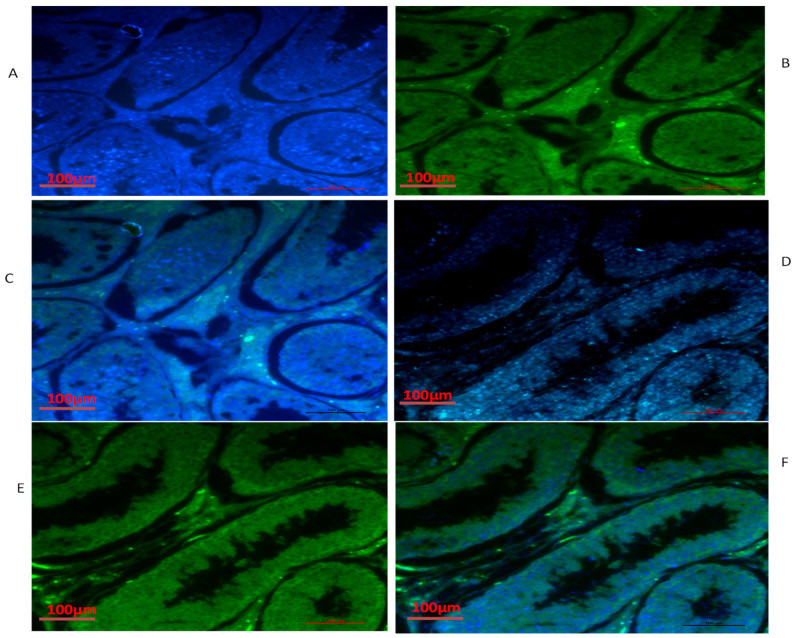
Immunofluorescence localization of TEX11 under various feeding conditions in yak testes: (**A**–**C**) natural grazing without concentrate supplementation; (**D**–**F**) natural grazing plus concentrate supplementation. Scale bar = 100 µm (**A**–**F**).

**Figure 7:**
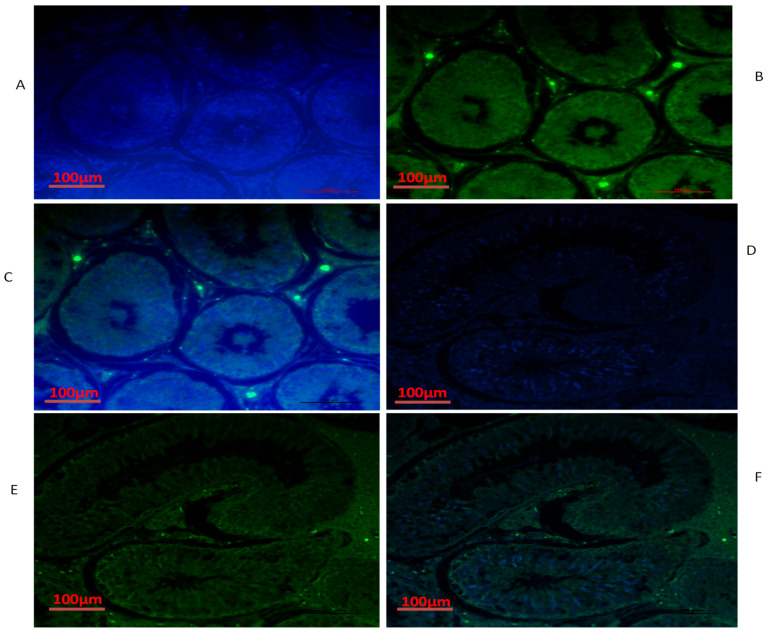
Immunofluorescence localization of BOLL protein in the testes of yaks under different feeding conditions: (**A**–**C**) natural grazing without concentrate supplementation; (**D**–**F**) natural grazing plus concentrate supplementation. Scale bar = 100 µm (**A**–**F**).

**Figure 8:**
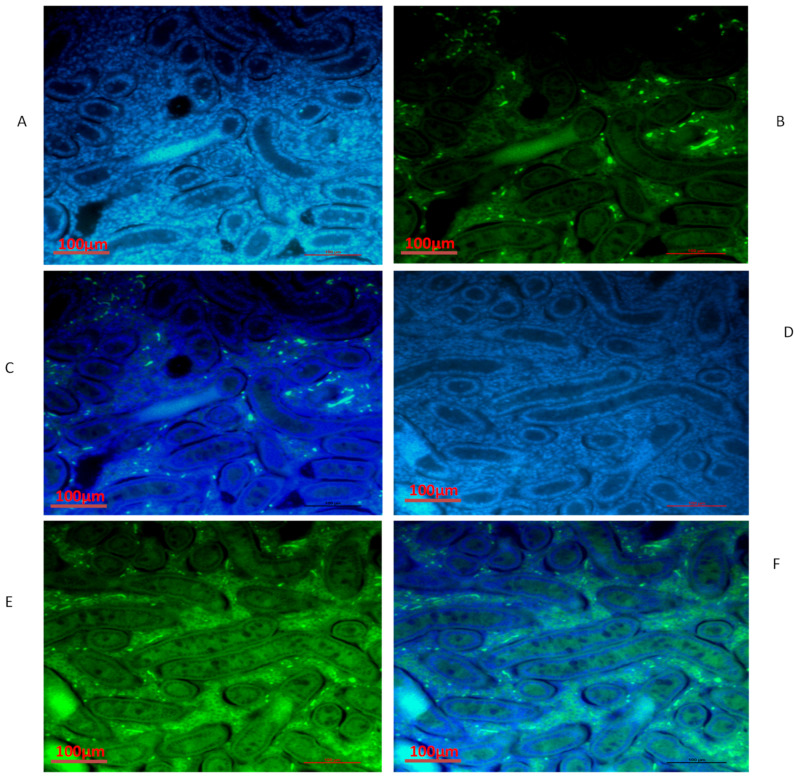
Immunofluorescence localization of ESRα protein in the testes of yaks under different feeding conditions: (**A**–**C**) natural grazing without concentrate supplementation; (**D**–**F**) natural grazing plus concentrate supplementation. Scale bar = 100 µm (**A**–**F**).

We also conducted immunofluorescence analysis in both groups (Figure 6, Figure 7 and Figure 8). Our immunofluorescence results showed that TEX11, BOLL, and ESRα were present in both groups, but the numbers of positive cells (yellow arrow) were higher in the tubules of the NG + CS group than in the group without concentrate supplementation. Additionally, the integrated optical densities of TEX11, ESRα, and BOLL were significantly higher in the NG + CS group than in the control group (*p* < 0.05).

### 3.5. Evaluation of Numerous Morphological Variations in the Natural Grazing without Concentrate Supplementation and Natural Grazing Plus Concentrate Supplementation Groups

**Figure 9:**
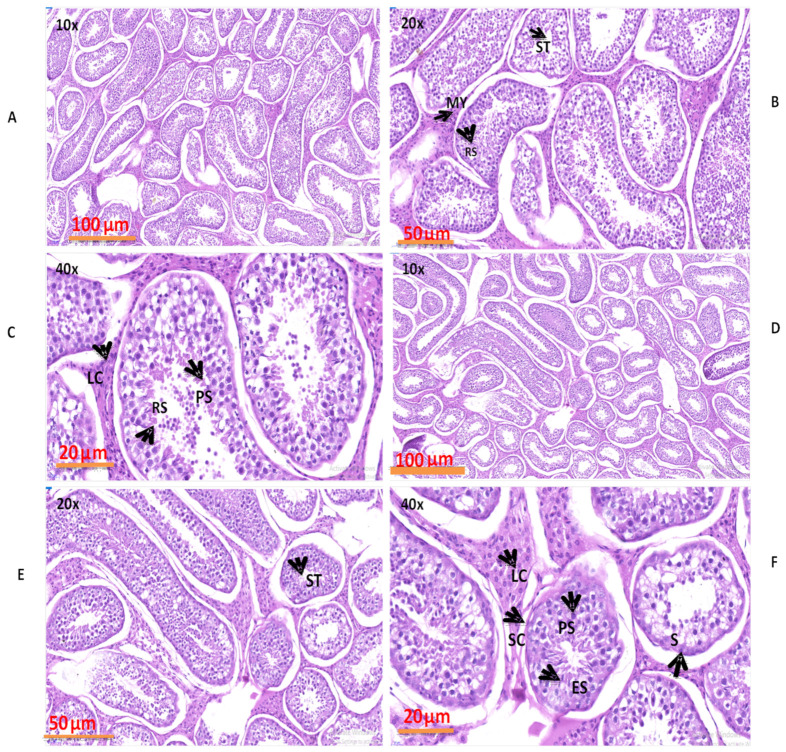
Morphological evaluations of yak testes under different feeding conditions: (**A**–**C**) natural grazing without concentrate supplementation; (**D**–**F**) natural grazing plus concentrate supplementation. Structures which were found: PS (primary spermatocyte), My (myoid cell), C (capillary), L (Leydig cell), ST (seminiferous tubule), S (spermatogonium), ES (elongated sperma).

The morphological results obtained by H&E staining are shown in Figure 9. These findings revealed that various structures, such as capillaries, Sertoli cells, primary spermatocytes, spermatogonia, round spermatids, myoid cells, and Leydig cells, were present in both groups. The seminiferous tubules, seminiferous epithelium, epithelial thickness, and volume densities were similar in both groups but higher in the NG + CS group as compared to NG group.

### 3.6. Spermatogenic Cells and Their Nuclei Diameters (μm) in Yak Testes

Our findings revealed that the diameters of spermatogenic cells and their nuclei increased after dietary energy supplementation (Table 3). In addition, the diameters of spermatogenic cells, Leydig cells, and their nuclei were significantly (*p* < 0.05) higher in the NG + CS group than in the control group. The diameters of the spermatids were not significant in either group (*p* > 0.05).

### 3.7. Diameters of Seminiferous Tubules and Numbers of Cells in the Testis

In our current study, the diameters of the seminiferous tubules increased significantly after dietary energy supplementation (Table 4). The diameters of the seminiferous tubules of the NG and NG + CS group were 225.30 ± 0.70 μm and 250.31 ± 0.44 μm, respectively. In addition, the height of the seminiferous epithelium was higher in the NG + CS group than in the NG group. Our findings demonstrate that the width of the tunica albuginea, the Leydig cell area, the luminal area, and the luminal diameter also increased when animals received dietary energy supplementation. In the present results, values for the volume density of seminiferous tubules were recorded from 672.10 ± 0.70% to 79.84 ± 0.40 ^b^% in the NG and NG + CS groups, and there was a significant difference between these groups. Furthermore, the total numbers of Leydig cells, spermatogonia, Sertoli cells, and spermatocytes per testis were lower in the NG group than in the NG + CS group.

## 4. Discussion

Nutrition plays a crucial role in maintaining the reproductive performance of animals. Previous findings have revealed that numerous physiological, nutritional, genetic, and environmental factors, including oxidative stress, seriously affect the production and reproductive potential of farm animals [37,38]. Since the 1980s, nutrition has been considered a crucial factor for the proper functioning of the reproductive system and sperm formation [39,40]. Presently, there is no research available on the influence of nutrition on the expression of reproduction-related genes. Hence, this study was designed to verify the effect of additional supplementation on the expression profiling of different genes related to reproduction.

*BOLL* mRNA plays a key role in spermatogenesis and maintaining testicular function, and BOLL homologs are also required for male fertility in mice and other species [18]. Another study revealed that improper *BOLL* expression was associated with a defect in meiotic maturation [41,42]. In addition, the knockout of *BOLL* homologs in several animal models, including mice, causes male sterility [43]. Similarly, our findings confirmed the presence of *BOLL* in the testes of yaks. We found that the concentrations of *BOLL* increased in yaks with increasing dietary energy levels. Likewise, testis-specific expression for *BOLL* mRNA has also been described in humans, pigs, mice [44], dairy goats [45], and chickens [46]. We also examined the expression of *BOLL* mRNA through qPCR in various tissues of the yaks (Figure 1). These findings revealed that the expression of *BOLL* mRNA was highest in the testis, followed by the epididymis and then the kidneys, in the NG + CS group. Another study found that *BOLL* expression was only observed in the testes of sheep but was not detected in other tissues [18]. Moreover, immunostaining and immunofluorescence results showed that BOLL protein was present in the testes of both groups, but the numbers of positive cells (yellow arrow) were higher in the tubules of the NG + CS group than in those of the control group. In addition, previous studies have reported that BOLL protein has been observed in round spermatids and spermatocytes of mice [45,46]. Similar outcomes have been described for the testicles of goats [47], Asian sea bass [48], and medaka [49]. Luetjens et al. [21] stated that BOLL proteins are constrained to spermatocytes in the testicles of normal adult men and they are missing in the testicles of infertile men. Interestingly, our Western blot results reported that the molecular weight of BOLL protein was 31 kDa in the testes and epididymides of Datong yaks. Our findings are in agreement with the results of Taotao et al. [18], who also detected 31 kDa bands for BOLL protein from sheep testes.

Estrogen plays a key role in the development of reproductive function and fertility [50,51]. However, the role of estrogen in male reproduction remains unclear. Hence, in the current study, we elucidated the role of *ESRα* in the testes of yaks. Our qPCR results illustrated that the expression of *ESRα* was lower in the tested and epididymides of the natural grazing group, while higher *ESRα* expression was observed in the testes and epididymides of the NG + CS group. Previous studies have shown that ESR1 and ESR2 are expressed in the fetal testis very early in development, and their distribution in various testicular cells has been extensively studied in mammals [14]. ESR1 has also been detected in the Sertoli cells of mature and immature rats [15]. The existence of ESR1 has also been documented in rat and human spermatozoa [52]. However, other studies have found ESR1 in Leydig cells [53,54]. Saunders et al. [55] did not find significant expression of ESR1 in the testes of humans. Moreover, our immunostaining and immunofluorescence results showed that ESRα proteins were present in the testes of both groups, but the number of positive cells (yellow arrow) was higher in the tubules of the NG + CS group than in the control group. O’Donnell et al. [56] showed that ESR1 is present in undifferentiated gonads and fetal Leydig cells until birth in rodents. Similarly, other studies have shown that ESR1 is expressed in the testes and epididymides of several species [16,17]. Immunohistochemistry studies have shown that ESRα is expressed in the ovaries and testicles of mullet (*Mugil cephalus*) [57]. Furthermore, Western blotting was performed to evaluate ESRα protein expression in the testes and epididymides of Datong yaks with and without concentrate supplementation (Figure 3). These results indicate that ESRα protein expression increased after concentrate supplementation. ESRα protein was lower in the testes and epididymides without concentrate supplementation than in the NG + CS group. On the other hand, Lin et al. [58] detected 56 kDa ESRα2 proteins and 67 kDa ESRα1 in Sertoli cells.

*TEX11* forms synaptonemal complexes on meiotic chromosomes and is vital for meiosis [58]. To confirm that *TEX11* is a testis-specific gene, we performed qPCR on various tissues of the yaks. Our qPCR results showed that TEX11 expression was higher in the testes and epididymides of the NG + CS group as compared to the control group. Previous findings have illustrated that *TEX11* is not localized in the lung, heart, brain, liver, skeletal muscle, kidney, and spleen, whereas it is highly expressed in the testicles of mature porcine animals [58]. Moreover, Wang et al. [8] reported that the *TEX11* transcript is present in germ cells at various spermatogenic stages, including type A and B spermatogonia, round spermatids, and meiotic spermatocytes. During meiosis, a high level of *TEX11* is upregulated from the preleptotene stage to the zygotene stage; however, its expression is downregulated in pachytene spermatocytes [11]. Additionally, our immunostaining and immunofluorescence results showed that TEX11 proteins were present in the testes of both groups, but the numbers of positive cells (yellow arrow) were higher in the tubules of the NG + CS group than in the control group. Another study revealed that *TEX11*-null mice may show elimination of spermatocytes and infertility [10]. In addition, a lack of *TEX11* reportedly led to reduced crossover formation and meiosis 1, although the males were fertile [59]. *TEX11* has also been identified in fetal oocytes, although female mutants were fertile with reduced litter size [60]. This expression pattern of yak *TEX11* confirmed that *TEX11* is indeed a testis-specific gene and is essential for male fertility.

Our H&E staining results illustrated that seminiferous tubule diameters increased significantly with dietary energy supplementation. The diameters of the seminiferous tubules in the NG and NG + CS groups were 225.30 ± 0.70 µm and 250.31 ± 0.44 µm, respectively. Previous findings presented the diameters of the seminiferous tubules in Nili Ravi buffalo bulls (176.8 ± 2.6 µm), Holstein bulls (223.44 µm), Simmental bulls (226.68 µm), and zebu bulls (246.6–257.9 µm) [61,62]. Furthermore, the height of the seminiferous epithelium was greater in the NG + CS (73.79 ± 0.60 µm) group compared to in the NG (65.70 ± 0.43 µm) group. Our results are in contrast with those obtained by Paulo et al. [63], who reported a seminiferous epithelium height of around 70.9 ± 2.2 µm in different zebu breeds. In most mammals, the seminiferous tubules are considered to be the main part of the testis, and their volume densities range between 70 and 90% of the testicular parenchyma [64,65]. Our results are in line with the above findings, and we reported the volume density of the seminiferous tubules in the NG (72.10 ± 0.70%) and NG + CS (79.84% ± 0.40%) groups. In addition, our findings demonstrated that the width of the tunica albuginea, the Leydig cell area, the luminal area, the luminal diameter, and the total number of Leydig cells per testis, spermatogonia, Sertoli cells, and spermatocytes were lower in the NG group than in the NG + CS group. This indicates that dietary modifications can be helpful in modulating male fertility.

## 5. Conclusions

In short, our H&E staining findings revealed that the diameters of the spermatogenic cells and seminiferous tubules and their nuclei increased after concentrate supplementation. Furthermore, qPCR showed that the expressional levels of *TEX11*, *ESRα*, and *BOLL* genes were higher in the NG + CS group than in the NG group. Additionally, our immunohistochemistry, immunofluorescence, and Western blot results showed that these proteins were lower in the testis and epididymis of the NG group than in the NG + CS group. Finally, it can be concluded that dietary energy supplementation can enhance the expression profiling of genes. Future studies will be conducted to investigate the specific molecular mechanisms of the *TEX11*, *ESRα*, and *BOLL* genes under different feeding conditions.

## Figures and Tables

**Table 1:** Ingredients and nutrient composition of the diets during the experiment.

Component	Grazing Group (Forage)	Concentrated Group
Protein	8.86 ± 0.58	28.39 ± 0.86
Calcium	3.73 ± 0.07	4.80 ± 0.05
Phosphorus	0.14 ± 0.03	0.17 ± 0.03
Neutral detergent fiber	60.45 ± 0.91	52.52 ± 0.09
Fat	0.89 ± 0.89	4.34 ± 0.15
Ash	8.97 ± 0.57	8.75 ± 0.07

**Table 2:** qPCR primers designed based on bovine genomic sequences.

Accession No.	Gene	Primers Sequence (5′ → 3′)	Product Length (bp)	Annealing Temperature (°C)
AY656813.1	ESRα	F: GCCATAGCCCATGCCTTTT R: TCATGGGGCTGTATGTGGTTT	748	60.33 59.64
NM_001168704.1	BOLL	F: AATAACCCAACGAGTGC R: AGTGACGAAGCCATACC	147	59.97 59.67
XM_024988612.1	TEX11	F: TCACGTCGGAGGAGCTTGAT R: AATTGCCGCAGGTGTGGTAG	145	55.00 55.00
NM_001034034.2:	GAPDH	F: AATGAAAGGGCCATCACCATC R: CACCACCCTGTTGCTGTAGCCA	204	55.85 60.00

**Table 3:** Spermatogenic cells and their nuclei diameters (μm) in the testes of yaks (mean ± SEM).

Germ Cells	NG	NG + CS
Spermatogonium	5.10 ± 0.60 ^a^	6.20 ± 1.00 ^b^
Spermatogonium nuclei	3.20 ± 1.10 ^a^	4.50 ± 1.20 ^b^
Spermatogonium nuclei	5.60 ± 0.70 ^a^	6.20 ± 0.90 ^b^
Primary spermatocyte	3.00 ± 1.00 ^a^	4.60 ± 1.18 ^b^
Primary spermatocyte nuclei	6.50 ± 1.69 ^a^	6.90 ± 1.30 ^a^
Round spermatid	6.89 ± 1.72 ^a^	7.01 ± 1.21 ^a^
Sertoli cells	4.50 ± 0.86 ^a^	5.10 ± 0.69 ^b^
Leydig cells	6.00 ± 1.19 ^a^	7.10 ± 1.55 ^b^

Means with different superscripts on the same row are significantly different (*p* < 0.05). NG = natural grazing without concentrate supplementation group; NG + CS = natural grazing plus concentrate supplementation group.

**Table 4:** Diameters of seminiferous tubules and numbers of cells in the testes of yaks under different feeding conditions (mean ± SEM).

Germ Cells	NG	NG + CS
Tubular diameter (μm)	225.30 ± 0.70 ^a^	250.31 ± 0.44 ^b^
Epithelial height (μm)	65.70 ± 0.43 ^a^	73.79 ± 0.60 ^b^
Luminal diameter (µm^2^)	74.90 ± 0.40 ^a^	85.62 ± 0.90 ^b^
Luminal area (µm^2^)	60.56 ± 4.60 ^a^	70.10 ± 6.38 ^b^
Leydig cell area (µm^2^)	134.43 ± 20.23 ^a^	140.76 ± 27.30 ^b^
Width of tunica	13.90 ± 1.00 ^a^	14.60 ± 0.50 ^a^
albuginea (µm)		
ST volume density (%)	72.10 ± 0.70 ^a^	79.84 ± 0.40 ^b^
Leydig cells (%)	27.20 ± 1.90 ^a^	29.50 ± 2.40 ^a^
Sertoli cells (%)	30.40 ± 1.63 ^a^	38.10 ± 2.70 ^b^
Spermatogonium (%)	175 ± 15.80 ^a^	190 ± 15.55 ^b^
Spermatocyte (%)	16.60 ± 1.40 ^a^	21.00 ± 1.60 ^b^

Means with different superscripts on the same row are significantly different (*p* < 0.05). NG = natural grazing without concentrate supplementation group; NG + CS = natural grazing plus concentrate supplementation group.

## Data Availability

The data presented in this study are available on request from the corresponding author.

## Supplementary Materials

The following are available online at https://www.mdpi.com/article/10.3390/biology10080731/s1, Figure S1 Western blotting of ESRα and BOLL proteins in the testes and epididymis of Datong yaks with and without concentrate supplementation.
