# Supplementary figures and images for: Expressional Profiling of TEX11, ESRα and BOLL Genes in Yak under Different Feeding Conditions
Source: Biology (Basel). 2021 Jul 30;10(8):731. doi: 10.3390/biology10080731 (PMC8389634; doi:10.3390/biology10080731)

Figure S1 Western blotting results

**ESR $\alpha$**

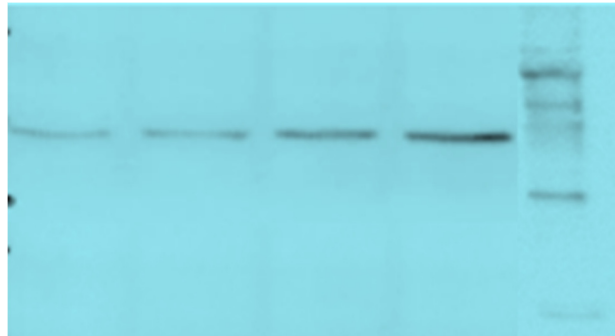

**BOLL**

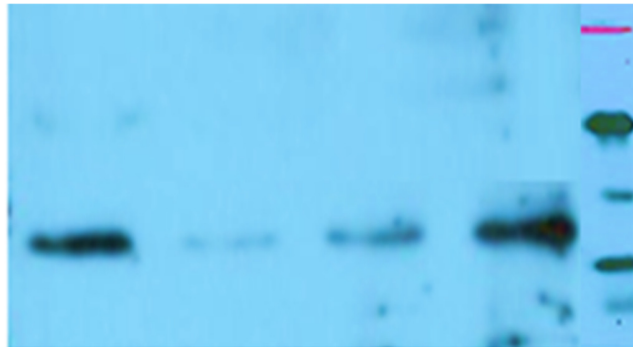

**$\beta$ -tubulin**

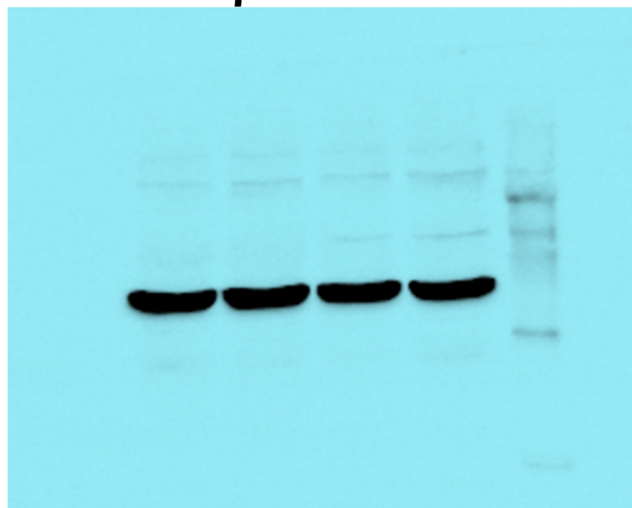

Supplement: Supplementary file 1 [file biology-10-00731-s001.zip › biology-1262290-supplementary.pdf]
